# The Final Step in Molybdenum Cofactor Biosynthesis—A Historical View

**DOI:** 10.3390/molecules29184458

**Published:** 2024-09-20

**Authors:** Ralf R. Mendel, Kevin D. Oliphant

**Affiliations:** Institute of Plant Biology, Technical University Braunschweig, Humboldtstraße 1, 38106 Braunschweig, Germany; k.oliphant@tu-braunschweig.de

**Keywords:** molybdenum, molybdenum cofactor, molybdenum insertase, nitrate reductase, gephyrin

## Abstract

Molybdenum (Mo) is an essential micronutrient across all kingdoms of life, where it functions as a key component of the active centers of molybdenum-dependent enzymes. For these enzymes to gain catalytic activity, Mo must be complexed with a pterin scaffold to form the molybdenum cofactor (Moco). The final step of Moco biosynthesis is catalyzed by the enzyme Mo-insertase. This review focuses on eukaryotic Mo-insertases, with an emphasis on those found in plants and mammals, which have been instrumental in advancing the understanding of Mo biochemistry. Additionally, a historical perspective is provided, tracing the discovery of Mo-insertase from the early 1960s to the detailed characterization of its reaction mechanism in 2021. This review also highlights key milestones in the study of Mo-insertase, including mutant characterization, gene cloning, structural elucidation at the atomic level, functional domain assignment, and the spatial organization of the enzyme within cellular protein networks.

## 1. Introduction

Molybdenum (Mo) has been recognized as a critical trace element in biological systems since its discovery in the early 20th century. Initial studies, including those conducted by Bortels [1] and Ter Meulen [2], brought to light the pivotal role of Mo in catalyzing nitrogen fixation in the bacterium *Azotobacter* and revealed its presence in various organisms, with notable concentrations in legumes and mammalian liver tissues. Despite these early findings, the broader significance of Mo and its utilization mechanisms across different life forms were not well understood then.

The understanding of the biochemical roles of Mo began to advance significantly with the investigation of the mammalian enzyme xanthine oxidase, which is particularly abundant in cow milk and liver [3]. By 1953, research had established Mo as an essential cofactor for the catalytic activity of xanthine oxidase [3]. This discovery paved the way for the identification of Mo in several other enzymes, such as aldehyde oxidase [4], nitrate reductase [5], and, later, sulfite oxidase [6]. Mo-dependent enzymes are now known to be ubiquitous across all domains of life, where they play crucial roles in various metabolic pathways, particularly those involving the transformations of nitrogen, sulfur, and carbon compounds [7]. The redox chemistry of Mo enzymes is notably complex, typically involving the transfer of two electrons and requiring the Mo atom to cycle between oxidation states IV and VI [7].

More than 50 Mo-containing enzymes have been characterized to date, with a majority identified in bacterial systems [8]. In contrast, only a handful of these enzymes have been found in eukaryotes, specifically five key enzymes: sulfite oxidase, xanthine oxidase, aldehyde oxidase, nitrate reductase, and mitochondrial amidoxime reductase (for review, see [7]). These eukaryotic Mo enzymes are involved in diverse and essential biological functions. Sulfite oxidase, for instance, is critical in the degradation of sulfur-containing amino acids and the detoxification of excess sulfite [9]. Xanthine oxidase plays a role in purine metabolism and the production of reactive oxygen species [10]. Aldehyde oxidase is versatile, oxidizing a variety of aldehydes and playing a key role in the biosynthesis of the plant stress hormone abscisic acid [11,12]. Nitrate reductase is essential in inorganic nitrogen assimilation in autotrophic organisms [13], and mitochondrial amidoxime reductase functions in detoxification processes [14].

In bacteria, well-studied Mo enzymes include formate dehydrogenase, nitrate reductase, DMSO reductase, xanthine dehydrogenase, and aldehyde oxidase [15]. Interestingly, in some extremophile bacteria and archaea, Mo is substituted by tungsten, a closely related transition element, which performs similar catalytic functions [16]. The diversity and adaptability of Mo and tungsten enzymes highlight the evolutionary versatility of these systems.

This review seeks to elucidate the complex processes by which Mo is made bioavailable and integrated into Mo-dependent enzymes within cells, focusing particularly on the function of molybdenum insertases (Mo-insertases). These specialized proteins catalyze within their active site the insertion of Mo into the dithiolene motif of MPT, thus forming Moco. We will also provide a historical perspective, highlighting the significant contributions made by studies on eukaryotic organisms, which have been drivers in advancing our understanding of Mo biochemistry.

## 2. Everything Began with Fungal Genetics

The foundational understanding of Mo biochemistry in biological systems began to take shape by studying fungal genetics. Mo, while required only in trace amounts, is crucial for organismal health, as its absence can be detrimental. The complexity of its role is underscored by the intricate cellular processes required to incorporate it into the active sites of enzymes. This necessity for detailed study led to the significant use of genetic approaches starting in the 1960s, particularly focusing on how cells acquire and utilize Mo. In various organisms, such as algae, fungi, and higher plants, the enzyme nitrate reductase (NR) is critical for the assimilation of inorganic nitrogen. In the filamentous fungus *Aspergillus nidulans*, research led by Pateman et al. [17] uncovered mutants deficient in NR, which also lacked the enzyme xanthine dehydrogenase. This simultaneous absence of two different Mo-dependent enzymes suggested the existence of a shared Mo-related cofactor, later termed the molybdenum cofactor (Moco) [17,18]. The genetic analysis revealed five distinct loci associated with this phenotype, named ‘cnx’ (cofactor for nitrate reductase and xanthine dehydrogenase). This discovery was pivotal, indicating that multiple genes were involved in the biosynthesis or utilization of Moco, contrasting with the single gene coding for nitrate reductase itself. The implications of this finding extended beyond fungi. In the 1970s, similar cnx-like mutants were identified in the plant *Nicotiana tabacum* [19], and, by the 1980s, analogous mutants were observed in the fruit fly *Drosophila melanogaster* [20]. Additionally, *Escherichia coli* strains exhibited similar mutant phenotypes, further supporting the notion that a series of gene products are essential to activate Mo biologically. These studies collectively established that the biological functionality of Mo is intricately tied to specific genetic components responsible for its processing and incorporation into enzymes. Mo is widely available in the environment, predominantly in the form of the molybdate anion, and is particularly abundant in ocean waters at concentrations around 100 nM [21]. In terrestrial environments, Mo can occupy various oxidation states, but it is the soluble molybdate form that is accessible to most eukaryotes and bacteria. The cellular uptake of molybdate is facilitated by highly specific transport proteins, which will be discussed later in this review. These transport systems are critical for ensuring the bioavailability of Mo for cellular processes, underscoring the complexity and specificity of Mo transport and utilization mechanisms.

## 3. The Discovery of Moco

The discovery of Moco marked a pivotal moment in understanding Mo biochemistry. Once molybdate enters the cell, it must be processed into Moco by a set of gene products. Multiple genetic loci are crucial for converting molybdate into a functional cofactor; yet, until the early 1970s, Moco remained a theoretical concept without a known chemical structure. The situation changed when Nason and colleagues [22,23,24] turned their attention to another filamentous fungus, *Neurospora crassa*, which had cnx-like mutants similar to those in *Aspergillus*.

In their groundbreaking work, Nason’s group provided the first biochemical evidence for a common cofactor shared among Mo-dependent enzymes. They extracted a low-molecular-weight fraction from denatured preparations of purified Mo enzymes derived from mammalian, plant, and bacterial sources. This fraction could fully reconstitute the inactive apoprotein of *Neurospora* NR, indicating the presence of a Mo-containing cofactor that could restore enzyme activity. The findings suggested that this ubiquitous Moco, with an obviously identical structure across different species, was an integral component of various Mo holoenzymes.

However, an important exception was noted with nitrogenase, another Mo-containing enzyme. Nitrogenase did not release Moco upon denaturation, unlike other Mo enzymes, indicating a distinct and unique cofactor specific to nitrogenase [25]. This differentiation highlighted the unique biochemical pathways involved in Mo enzyme function, suggesting specialized roles and structures for different Mo cofactors within biological systems. This discovery laid the foundation for further research into the specific mechanisms and structural characteristics of Moco and its role in various enzymatic processes.

## 4. The Chemical Nature of Moco

The chemical elucidation of Moco was a significant advancement in understanding its role in biological systems. This breakthrough came through the work of J. Johnson and K.V. Rajagopalan in the early 1980s. They utilized spectroscopic analyses on Moco extracted from chicken and rat liver tissues, which revealed the presence of a pterin component [26]—a key discovery in defining Moco’s structure. The challenge in studying Moco lies in its instability; it is highly labile and prone to rapid oxidation when exposed to air. Consequently, researchers had to rely on the degradation and oxidation products of the cofactor to further their studies.

**Figure 1:**
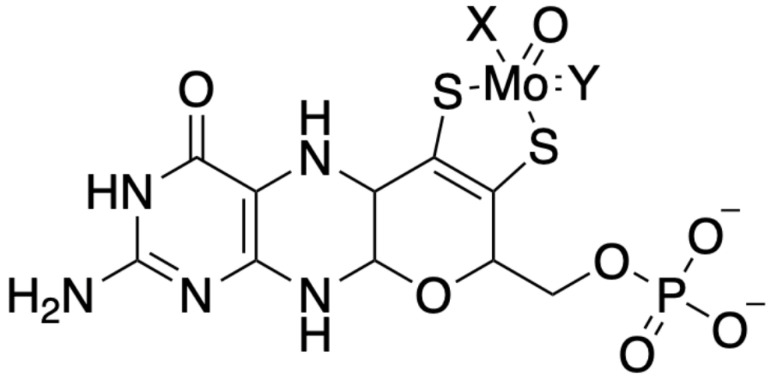
Moco in eukaryotic Mo enzymes. Eukaryotic Mo enzymes are classified into two distinct families. In the sulfite oxidase family, the X position is occupied by single-bonded sulfur from a cysteine residue within the protein. The Y position is occupied by double-bonded oxygen. Conversely, X is represented by a double-bonded inorganic sulfur in the xanthine oxidase family, while Y is a hydroxyl group.

Under acidic conditions, the oxidation of Moco produced fluorescent pterin derivatives, which were instrumental in uncovering the cofactor’s structure. These studies led to the proposal that Moco consists of a 6-alkylpterin moiety. This structure includes a 4-carbon side chain, which features an enedithiolate group at the C1’ and C2’ positions and a terminal phosphate ester group (as illustrated in Figure 1) [27]. The detailed structural characterization of Moco clarified its chemical nature and provided insights into its functional roles in Mo-dependent enzymes across different organisms. Understanding Moco’s structure has been crucial for deciphering its function in various enzymatic processes, including its involvement in electron transfer and catalysis within the active sites of enzymes.

In the late 1990s, the detailed crystallographic structures of Mo enzymes [28] provided critical insights into the core structure of Moco. These studies confirmed the presence of a third pyrano ring, which forms between the hydroxyl group at the C3’ position of the side chain and the C7 atom of the pterin in its 5,6-dihydro state. The formation of this pyrano ring results in a fully reduced, hydrogenated pterin. The unique nature of the pterin component in Moco led to the designation of the metal-free form of the cofactor as molybdopterin, or metal-binding pterin (MPT). This nomenclature reflects MPT’s ability to coordinate not only Mo but also tungsten, another transition metal that can replace Mo in certain bacterial enzymes. Notably, Moco can exist in bacteria as a dinucleotide derivative, where the MPT moiety is associated with nucleotides such as GMP or CMP [15].

The pterin moiety of Moco plays several crucial roles within Mo enzymes. Firstly, it helps correctly position the catalytic metal within the enzyme’s active site. The nature of the ligands attached to Mo can vary depending on the enzyme class, influencing the enzyme’s specific function [7]. Secondly, the pterin moiety may modulate the redox properties of the Mo atom, which is crucial for the enzyme’s catalytic activity. Thirdly, the pterin structure may facilitate electron transfer to and from the Mo center, leveraging its delocalized electrons. Finally, the various reduction states and structural conformations of the pterin may assist in channeling electrons to or from other prosthetic groups within the enzyme complex [7].

Structural analyses, particularly through X-ray crystallography, revealed that Moco is not positioned on the enzyme’s surface but is deeply embedded within the protein [29,30,31]. This deep positioning is facilitated by a tunnel-like structure, which allows substrates and other interacting prosthetic groups to access the cofactor. This configuration is vital for the proper function of Mo enzymes, ensuring that the reactive center is shielded and that electron transfer processes are efficiently managed. Those structural insights underscore the sophisticated nature of Moco and its essential roles in various biochemical pathways across different organisms.

## 5. The Gene for Mo-Insertase Was the First Eukaryotic Gene Cloned for Moco Biosynthesis

The detailed biochemical characterization of NR-deficient mutants has been instrumental in advancing our understanding of the biochemistry and genetics underlying Moco biosynthesis, including bacteria, fungi, and plants. These mutants uniformly exhibited a lack of NR activity, indicating disruptions in the Moco biosynthesis pathway. A practical initial approach was cultivating these mutants on a high-molybdate medium (1 mM) to identify potential molybdate-repairable phenotypes. Among the *A. nidulans cnx* mutants, one locus was partially repairable with molybdate [32]. Similarly, in the plant *N. tabacum*, certain mutants displayed partial molybdate-repairable characteristics [33], as did some mutants among the newly described *N. crassa nit* loci [34]. It was suggested that the gene products of these molybdate-repairable loci likely play a role in transferring or inserting Mo into molybdopterin (MPT), a key late step in Moco biosynthesis. This hypothesis was later validated through molecular analyses, leading to the identification of Mo-insertase enzymes. Further comprehensive analyses of Moco biosynthesis mutants in *E. coli* and *N. tabacum* led to the establishment of a working model for Moco biosynthesis published in 1992 [35,36].

As the MPT structure was conserved across all organisms, it was tempting to postulate a potentially similar biosynthetic pathway in diverse life forms, including humans and plants. The sequencing of the *E. coli* genome in 1997 [37], followed by the human [38] and *Arabidopsis thaliana* [39] genomes in 2000, provided a deeper understanding of the genetic components involved. However, research into Moco biosynthesis in these organisms began before the genomic era, with significant early insights coming from studies on plants, particularly through the detailed characterization of NR-deficient mutants using callus cultures and in vitro plants. Our group chose to name them *cnx’* [40], similar to the *Aspergillus* geneticists’ suggestion [18]. This pre-genomic work laid the foundation for understanding the genetic and biochemical pathways of Moco biosynthesis, with the plant *A. thaliana* serving as a key model organism.

**Figure 2:**
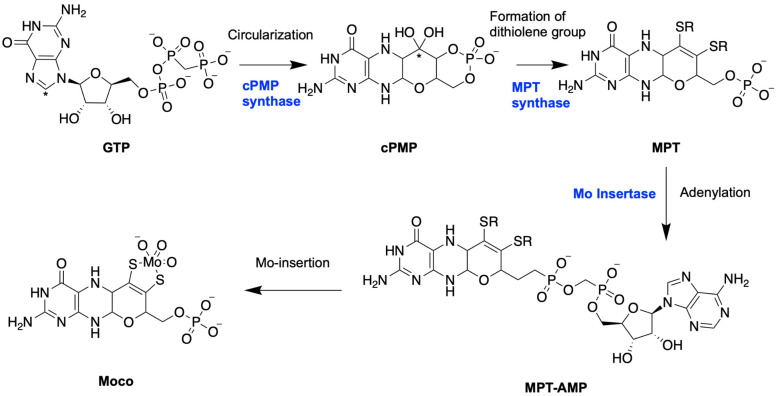
Moco biosynthesis pathway in eukaryotes. The pathway is divided into four distinct stages. During step 1, the C8 atom of guanosine triphosphate (GTP), marked with a star, is incorporated by the cPMP synthase between the 2′ and 3′ carbon atoms of ribose, forming the new C1’ position in the pterin’s four-carbon side chain, which is similarly starred in cPMP. In the second step, a dithiolene motif is inserted by the MPT synthase. Steps three and four are catalyzed by specific domains of the Mo-insertase in which the G-domain adenylates MPT, and then molybdate is inserted by the E-domain.

In 1995, our group cloned the first eukaryotic gene involved in Moco biosynthesis from *A. thaliana* [41]. This gene corresponded to the molybdate-repairable mutant, and the consortium for the nomenclature of plant genes designated it as Cnx1, reflecting its well-characterized role in the biosynthesis process [42]. Subsequent studies confirmed that Cnx1 catalyzes the final step in Moco biosynthesis, highlighting its essential role. The conserved pathway of Moco biosynthesis involves four key steps, as shown in Figure 2 [43]: (1) the conversion of GTP to cyclic pyranopterin monophosphate (cPMP), (2) the conversion of cPMP to MPT, (3) the adenylation of MPT by Mo-insertase Cnx1 to yield MPT-AMP, and (4) the insertion of Mo into MPT-AMP by the multifunctional Cnx1 enzyme, resulting in the formation of active Moco. This is a simplified overview of the functioning of Mo-insertase. Later, we will describe it in detail.

## 6. Domain Organization of Mo-Insertases

**Figure 3:**
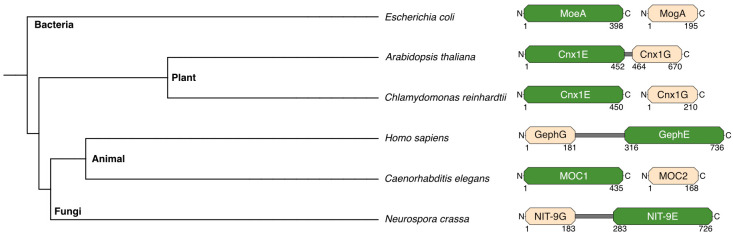
Orientation of the Mo-insertase domains across species. This figure presents a phylogenetic tree generated using data from the NCBI taxonomy database, showcasing selected species from various kingdoms. In eukaryotes, the E- and G-domains are organized within a single protein, while in most prokaryotes, these domains are expressed separately, with two noted exceptions (*C. reinhardtii* and *C. elegans*). The schematic representation highlights the G-domain in beige and the E-domain in green, with domain boundaries annotated by their start and end residue numbers. Species include *E. coli* [41]. *A. thaliana* [45], *C. reinhardtii* [46], *Homo sapiens* [47], *C. elegans* [48], and *N. crassa* [49].

The cloning of the *cnx1* gene was achieved using functional complementation, a crucial technique at the time, given the lack of sequence databases and internet-based search tools. Our group isolated *cnx1* by complementing the molybdate-repairable *E. coli mogA* mutants [41], highlighting the evolutionary conservation of this protein’s function. Surprisingly, the *cnx1* gene encodes a protein significantly larger than MogA. Cnx1 consists of two distinct domains (Figure 3): the N-terminal domain (we named it E-domain) is homologous to *E. coli* MoeA, and the C-terminal domain (we named it G-domain) is homologous to MogA. This structure was unexpected but provided insights into the evolutionary conservation and specialization of Mo-insertases. The G-domain of Cnx1, even when expressed separately, can effectively substitute MogA’s function when expressed in *E. coli* [44], demonstrating a high degree of functional conservation. In contrast, the E-domain does not replicate the function of bacterial MoeA, indicating some divergence in function.

With the availability of comprehensive genomic data from various model organisms, Mo-insertases can be categorized based on their domain arrangements (Figure 3). In plants, the E-domain is positioned at the N-terminus, while in animals and fungi, the G-domain is at the N-terminus. Prokaryotes like *E. coli* typically express these domains as separate proteins. Exceptions include the alga *Chlamydomonas reinhardtii* and the nematode *Caenorhabditis elegans*, which also express the domains separately. This diversity in domain organization reflects the evolutionary adaptations and functional needs of different organisms, underscoring the versatility and importance of Mo-insertases in various biological contexts.

### 6.1. Assigning Functions to the Mo-Insertase Domains

The functional characterization of Mo-insertases has predominantly been conducted using the Arabidopsis Cnx1 protein as a model. This review will thus focus on the insights gained from studying Cnx1.

#### 6.1.1. Cnx1 G-Domain

For a protein to act as a Mo-insertase, it must bind both substrates—Mo and MPT—to facilitate the conversion of MPT into active Moco. Thus, our group purified the two domains of Cnx1 separately and showed a high-affinity interaction between MPT and the Cnx1 G-domain (with a dissociation constant Kd of 100 nM) [44]. The Cnx1 E-domain also exhibited the ability to bind MPT, albeit with lower affinity (Kd of 1 µM). These findings suggested a common binding motif for MPT within both domains of Cnx1.

**Figure 4:**
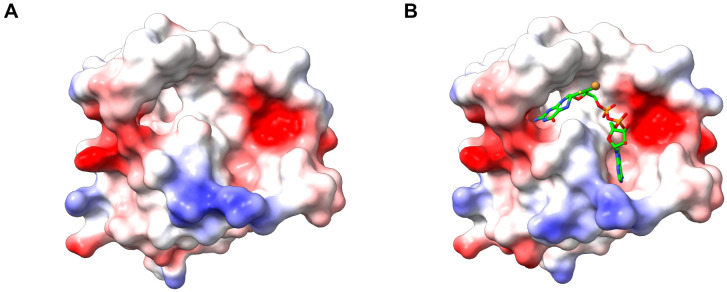
Structural elucidation of the Cnx1 G-domain. (**A**) The electrostatic surface representation of the first crystal structure of CNX1G (PDB: 1EAV) was resolved in 2001 [50]. (**B**) The electrostatic surface representation of the first crystal structure of CNX1G in complex with MPT-AMP (PDB: 1UUY) resolved in 2004 [52]. The applied color scheme is carbon (green), oxygen (red), nitrogen (blue), phosphorus (orange), sulfur (yellow). The surfaces of both structures are color-mapped according to the Coulombic electrostatic potential, ranging from red, indicating negative potential, through white to blue, representing positive potential.

Further investigations, including mutagenesis screening, indicated that MPT-binding was not the sole function of the G-domain. Interestingly, the G-domain, but not the E-domain, could complement the function of the molybdate-repairable *E. coli* mutant *mogA*, leading to the initial assumption that the G-domain was responsible for inserting Mo into MPT. Subsequent crystallization of the Cnx1 G-domain revealed an empty cavity, suggesting a potential site for substrate binding and catalysis [50]. Earlier, the structure of *E. coli* MogA (=the homolog of the G-domain) was solved, but also with an empty cavity [51]. It was only through structure-guided mutagenesis that the role of the G-domain was clarified. This approach identified MPT-AMP as a novel intermediate bound within the cavity three years after the initial crystallization attempts [52] (Figure 4). This discovery shifted the understanding of the G-domain’s function: rather than directly inserting Mo into MPT, the G-domain’s primary role is to bind MPT and convert it into MPT-AMP, also known as adenylyl MPT. Although the term “adenylated MPT” was used in the original publication in 2004 and is commonly found in the literature, the term adenylyl MPT is biochemically precise. However, we will adhere to the original old terminology throughout the review. The identification of MPT-AMP as a critical intermediate in Moco biosynthesis has provided significant insights into the mechanistic steps involved in this pathway and the specific roles played by the Mo-insertase domains.

#### 6.1.2. Cnx1 E-Domain

**Figure 5:**
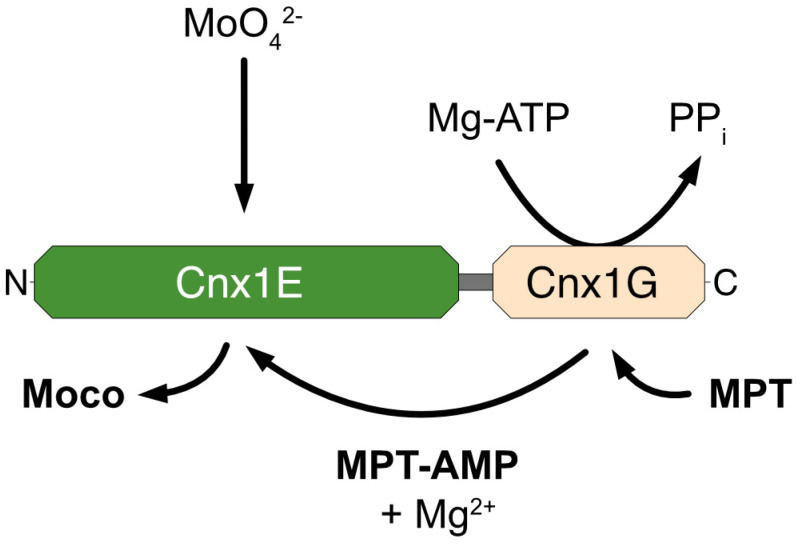
Functional schematic of Mo-insertase. This figure presents a detailed schematic model illustrating the function of the Mo-insertase enzyme in the biosynthesis of Moco. The model highlights the stepwise process by which the Mo-insertase facilitates the insertion of Mo into the pterin-based precursor MPT, forming active Moco essential for the catalytic function of various Mo-dependent enzymes. Key intermediates, domains, and molecular interactions involved in this process are depicted. For details, see the text.

Above, we described that both domains of Cnx1 were shown to bind MPT [44]. As the function of the G-domain was to generate MPT-AMP, we assumed the function of the E-domain should be to receive MPT-AMP from the G-domain, to hydrolyze the phosphoanhydride bond and to insert molybdate into MPT, thus forming Moco (Figure 5).

**Figure 6:**
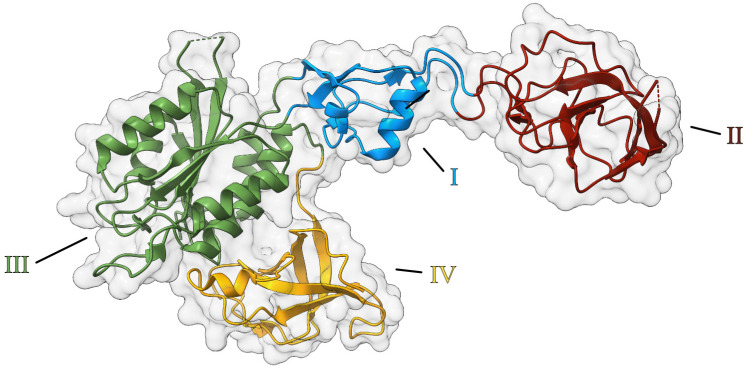
Structure of the Cnx1 E-domain. The structure of a CNX1E monomer is displayed in a cartoon representation with a transparent surface. CNX1E was crystallized with a single monomer in the asymmetric unit, with the physiological dimer formed through crystallographic symmetry (PDB: 5G2R) [54]. The four subdomains are color-coded as follows: domain I in blue, domain II in red, domain III in green, and domain IV in yellow.

Two primary questions arose concerning the function of the E-domain: (1) Does molybdate act as a direct donor for the insertion of Mo into MPT, or must it undergo some form of processing before insertion? (2) What is the role of AMP in this process? Initially, it was proposed that AMP played a role in generating an AMP-activated molybdate species, serving as a transient intermediate before insertion into MPT [52,53], and this idea persisted for about a decade in the literature, until 2017, when we published the atomic structure of the Cnx1 E-domain [54] (Figure 6).

**Figure 7:**
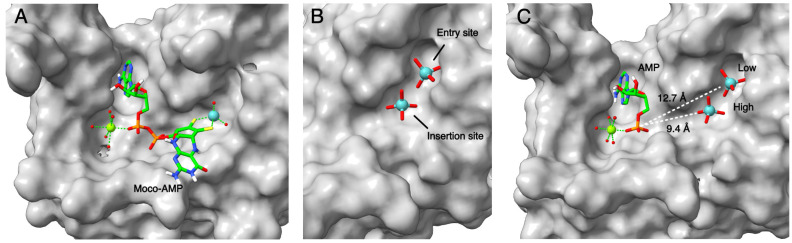
Cnx1 E-domain interaction with Moco. (**A**) Oxo-anion-binding pocket of CNX1E from the S269D, D274S variant in complex with Moco-AMP (PDB: 6Q32). (**B**) Positioning of Mo in the entry site and the insertion site in the wild-type CNX1E structure (PDB: 6ETF) with the entry site superimposed from the S238A mutant structure (PDB: 6GB4). (**C**) Depiction of AMP and the measured distance between the Mo atom and the phosphate group. High and low denote the high-occupancy site and the low-occupancy site of Mo, respectively. Ligands in panels (**A**–**C**) are represented as ball and stick or spheres, including the magnesium–water complex. The applied color scheme is carbon (green), oxygen (red), nitrogen (blue), phosphorus (orange), sulfur (yellow), magnesium (light green), hydrogen (white), and molybdenum (teal). Adapted from Probst et al., 2021 [57] and Krausze et al., 2018 [56].

Cnx1E was dimeric in solution and in the solid crystal state [54]. The monomer is an elongated molecule with four subdomains (I–IV), and subdomain III was found to be highly similar to the Cnx1 G-domain. Two decades earlier, the structure of the bacterial homolog MoeA was published [55], revealing an identical subdivision in four subdomains. In the Cnx1 E-domain, the high similarity of subdomain III to its G-domain pointed to the binding site of MPT-AMP and where the Mo insertion should take place. We suggested AMP serve as an anchor for positioning MPT in an orientation ready for Mo insertion. In subsequent work [56], we identified two mutually exclusive binding sites for molybdate in the E-domain (Figure 7): an initial binding site from which molybdate moves forward to the actual insertion site, facilitated by a minor structural change in the active site of the E-domain.

## 7. Mechanism for Molybdate Insertion

**Figure 8:**
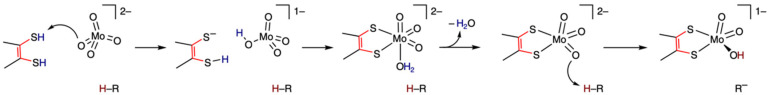
Mechanism of molybdate insertion into MPT-AMP. Details are given in the text (modified after Probst et al., 2021 [57]).

Based on the structure-guided generation, crystallization, and biochemical characterization of amino acid exchange variants of the Cnx1 E-domain, the following mechanism was suggested for inserting Mo into MPT-AMP (Figure 8). This was only possible by the joining of Marty Kirk (Albuquerque, NM, USA) as a specialist in advanced X-ray absorption spectroscopy [57]. We must remember that molybdate has four oxygen atoms, while in Moco, the Mo center has only three oxygens. (1) Molybdate binds to its initial binding site in the E-domain, opposing the dithiolene moiety of MPT-AMP. (2) Molybdate moves forward to the insertion site. Here, one of its oxygens becomes protonated by the dithiolene moiety of MPT. Thus, formed water should serve as a leaving group. (3) The now-formed Mo-center becomes protonated by one of the active site residues, thus preventing the back-reaction. (4) Freshly formed Moco is still in bond with AMP, which we described as an unexpected novel and final intermediate in Moco biosynthesis. (5) Cleavage of the Moco-AMP phosphoanhydride bond and liberation of mature Moco from anchoring AMP. Calculations of the energy indicate that once MPT-AMP and molybdate are in their correct positions within the active center, the two protonations of molybdate proceed, nearly barrierless [57]. More details are given in [58]. Finally, this reaction sequence showed that the early postulate of an AMP-activated molybdate species does not hold true.

### 7.1. The Role of Magnesium Ions

**Figure 9:**
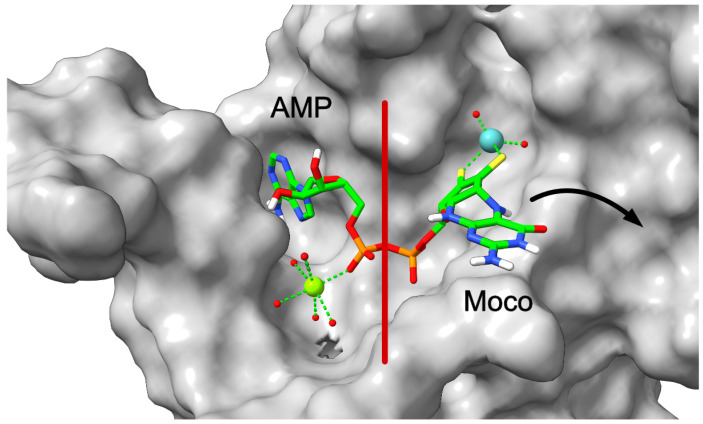
Moco release mechanism facilitated by the Cnx1 E-domain. Moco is released from the active site following Mo insertion into the dithiolene motif, with AMP and Mg²⁺ remaining briefly within the active site. The red line highlights the phosphate bond cleavage site after Mo insertion. This representation utilizes the S269D, D274S variant in complex with Moco-AMP (PDB: 6Q32). The color scheme is carbon (green), oxygen (red), nitrogen (blue), phosphorus (orange), sulfur (yellow), magnesium (light green), hydrogen (white), and molybdenum (teal).

Early on, structure-based work identified magnesium ions in the active site of MoeA [59], and consistently, we found them in Cnx1 E-domain structures [52,57]. Functional in vitro experiments showed a requirement for magnesium as well [60]. In an analogy to the mechanism of cleaving nucleosides [61], we assume that one of the five water molecules seen in the crystal structures coordinated by the magnesium ion is starting a nucleophilic attack of the phosphate, thus hydrolyzing the Moco-AMP phosphoanhydride bond and releasing Moco (Figure 9). This process should be initiated by a conformational change in Moco-AMP [57].

### 7.2. The Human Mo-Insertase Gephyrin

In the late 1990s, our research shifted towards understanding human Moco biosynthesis, and we identified Gephyrin as the mammalian counterpart to the plant Cnx1 Mo-insertase. Gephyrin was already known as a structural protein that anchors glycine receptors to the cytoskeleton at synapses, playing a crucial role in forming glycinergic synapses [62,63]. However, based on its homology to Cnx1, we proposed an additional role for Gephyrin in Moco biosynthesis. This hypothesis was confirmed when experiments demonstrated that recombinant Gephyrin could bind MPT with high affinity and restore Moco biosynthesis in various systems, including *E. coli*, plants, and mammalian cell lines. Moreover, inhibiting Gephyrin expression significantly reduced Moco content in cultured murine cells [64]. Gephyrin knockout mice lacked synaptic glycine receptor clustering and exhibited symptoms akin to Moco deficiency [65], underscoring Gephyrin’s dual function: one in Moco biosynthesis and another in receptor clustering.

Interestingly, in Gephyrin, the arrangement of the functional domains is reversed compared to Cnx1, with the G-domain at the N-terminus and the E-domain at the C-terminus. This domain orientation is also found in the Gephyrin-homologous protein Cinnamon in *Drosophila melanogaster* [66] and in the fungal protein *N. crassa* Nit-9 [67], suggesting two independent evolutionary events that shaped the structure of Mo-insertases in different lineages. Additionally, Gephyrin features a notably longer linker region between its E- and G-domains compared to Cnx1 (134 vs. 11 amino acids, Figure 3). This linker region has been implicated in modulating signaling pathways and is subject to palmitoylation, a modification essential for Gephyrin’s association with the postsynaptic membrane [68].

The evolution from bacterial monofunctional proteins to multifunctional eukaryotic proteins like Gephyrin highlights an adaptive process potentially aimed at improving substrate–product channeling and the efficiency of complex biosynthetic pathways. 

### 7.3. Mo-Insertases Bind to the Cytoskeleton and Anchor a Putative Moco-Biosynthesis Multiprotein Complex

Gephyrin, known for its role in anchoring neuroreceptors, links these receptors to actin filaments of the cytoskeleton via its E-domain [63]. This function is not unique to Gephyrin, as its plant homolog Cnx1 also exhibits cytoskeleton binding properties, albeit in the context of Moco biosynthesis, as shown by Günter Schwarz [69]. The cytoskeleton binding of Cnx1 was first demonstrated in vitro [69] and further confirmed in vivo through live-cell imaging using confocal laser scanning microscopy by our group [70,71]. This binding led to the hypothesis that the anchoring might play a crucial role in stabilizing a putative multienzyme protein complex involved in Moco biosynthesis. Additionally, it was speculated that such anchoring could position Cnx1 near a molybdate importer, thus facilitating efficient sequestration of incoming molybdate into the molybdate insertase Cnx1 [69].

**Figure 10:**
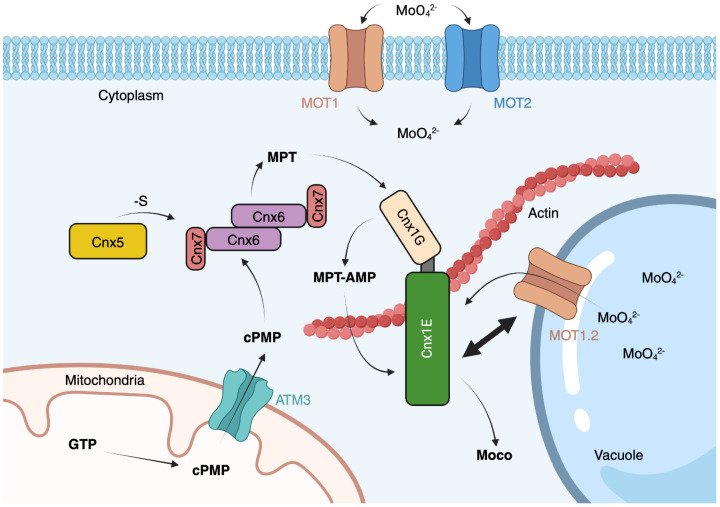
Moco homeostasis in *A. thaliana*. The entire Moco biosynthetic pathway, from GTP to Moco, is depicted alongside the enzymes involved at each stage. Distinct proteins catalyzing the biosynthesis steps are color-coded for clarity. All known intermediates are sequentially presented across the four key steps of Moco synthesis. The MOT1 and MOT2 proteins facilitate the transport of the molybdate anion into the cytosol, while MOT1.2 mediates Mo transport from the vacuole to the Mo-insertase Cnx1. Prior research has demonstrated interactions between these proteins, as indicated by the two-sided arrow [72]. GTP is converted to cPMP by the Cnx2 and Cnx3 proteins, with the mitochondrial ABC transporter ATM3 shuttling cPMP to the cytosol. MPT-synthase, composed of Cnx6 and Cnx7, transforms cPMP into MPT, with Cnx5 supplying the sulfur. The Cnx1 G-domain activates MPT, converting it into MPT-AMP, which is then transferred to Cnx1E. Finally, Cnx1E incorporates Mo into MPT-AMP and removes AMP to generate the active Moco. For detailed descriptions, refer to the main text.

Further research using bimolecular fluorescence complementation of split fluorescent proteins provided evidence supporting the existence of a multiprotein complex for Moco biosynthesis. Within the living plant cell, we observed tight protein–protein interactions among Cnx5, Cnx6/7, and Cnx1, which catalyze steps 2 and 3 of the biosynthesis pathway [70]. This complex formation likely plays a crucial role in channeling the fragile intermediates of Moco biosynthesis, protecting them within a transient multiprotein assembly. Such a spatial organization would facilitate the efficient processing of these intermediates and also underscore the functional significance of the cytoskeleton anchoring in the overall Moco biosynthetic pathway (see Figure 10).

### 7.4. Plant Mo-Insertase Cnx1 Binds to Molybdate Transporters

Molybdate uptake into the cell is facilitated by highly specific transport systems that fall into two groups. MOT1 transporters are predominantly found in plants, algae, and fungi, where they are localized in the plasma membrane and the vacuolar membrane [49,72,73]. In contrast, MOT2 transporters are found in the plasma membrane of most eukaryotes, including animals and plants [72,74]. MOT2 imports molybdate into the cell, which is immediately sequestered through Cnx1 into Moco biosynthesis as the main user of Mo. As Mo is an essential micronutrient, plants also store it temporarily in their vacuole, where we found MOT1.2 in the vacuolar membrane [73], serving as a molybdate exporter [75]. It is suggested that molybdate enters the vacuole in the form of a glutathione complex through a general heavy metal import mechanism [75]. We developed a FRET-based probe to analyze the Mo homeostasis [76]. Physiologically, it makes sense that Cnx1 also has protein contact with this transporter, which is an additional molybdate supplier for Moco biosynthesis.

## 8. Is There Life without Molybdenum?

All higher organisms possess Mo enzymes, yet genome-wide database analyses have revealed that many bacteria and unicellular lower eukaryotes, such as yeasts, fungi, and protists, do not require Mo. This observation suggests that Mo utilization is an ancient trait, once common across almost all species in the three domains of life. It is believed that Mo utilization was present in LUCA, the last universal common ancestor of life [77].

Large-scale comparative genome analyses have shown that approximately 30% of eukaryotic species, primarily unicellular organisms like yeasts, fungi, and protists, have lost the ability to utilize Mo [78]. This loss is highly correlated with specific habitat and lifestyle adaptations, particularly among obligate intracellular parasites and symbionts. These organisms tend to have condensed genomes due to the evolutionary pressure to minimize genome size due to their parasitic lifestyle. Despite losing their own Mo enzymes, these organisms may still benefit from Mo-dependent enzyme functions provided by their hosts. In contrast, all multicellular eukaryotes retain a dependence on Mo [79].

Interestingly, model yeasts such as *Saccharomyces cerevisiae* and *Schizosaccharomyces pombe* do not play a role in Mo research because they lack Mo enzymes. However, other yeasts, such as *Pichia pastoris*, do require Moco. Evidence suggests that *Saccharomyces cerevisiae* may have once possessed the machinery for Moco biosynthesis. Specifically, a protein sequence homologous to MoeB, an enzyme involved in Moco biosynthesis, is found in the highly conserved ubiquitin-activating enzyme E1 (Uba4) in eukaryotes. This sequence similarity implies that E1 could have evolved from an ancestral *moe*B gene [80], which in *Saccharomyces cerevisiae*, along with the entire Mo metabolism, was lost during evolution.

## 9. Outlook

During the evolution from prokaryotes to eukaryotes, the pterin core structure of Moco has not changed. Accordingly, the basic steps of its biosynthetic pathway are conserved. However, some notable differences between prokaryotes and eukaryotes took place during evolution. The Mo-insertase Cnx1 and animal Gephyrin are examples where single monofunctional prokaryotic proteins (comprising *E. coli* MogA and MoeA) have been fused to form two-domain multifunctional eukaryotic proteins. What is the driver for this evolutionary process? It was assumed that this fusion might facilitate better substrate–product channeling [81]. However, in the alga *C. reinhardtii* and the nematode *C. elegans,* this fusion did not happen. The length of the linker between the two domains is very variable between organisms, and our experiments show that it is also sequence-specific and orientation-dependent (unpublished). Furthermore, *C. elegans* can take up Moco from its diet, partially removing the necessity for its endogenous biosynthesis [82]. Thus, the linker is not simply a spacer but should be regarded as a linkage domain. In mammals, the linker (134 amino) functions as a separate domain for membrane association [68].

Mo-insertases are multifunctional proteins that, beyond their catalysis of Mo-insertion into MPT, fulfill additional tasks important for the cell: They bind to the cytoskeleton as well as to Mo-transporters, they anchor the Moco biosynthesis complex, and they facilitate the sequestering of incoming molybdate into Moco. As Moco is very fragile, Moco is generally believed to be permanently protein-bound within the cell. Therefore, Mo-insertases should interact with a whole set of downstream Moco-binding proteins, either Moco-user enzymes or Moco-binding proteins belonging to the insertion-competent Moco pool within the cell.

Further experiments will shed light on the evolutionary dynamics and significance of Mo-insertase diversity, underscoring the importance of this group of enzymes.

## Data Availability

Data are contained within the article.

## References

[1] Bortels H. (1930). Molybdän als Katalysator bei der biologischen Stickstoffbindung. Arch. Mikrobiol..

[2] Ter Meulen H. (1932). Distribution of molybdenum. Nature.

[3] Richert D.A., Westerfeld W.W. (1953). Isolation and identification of the xanthine oxidase factor as molybdenum. J. Biol. Chem..

[4] Mahler H.R., Mackler B., Green D.E. (1954). Studies on metallo-flavoproteins.III. Aldehydeoxidase: A molybdoflavoprotein. J. Biol. Chem..

[5] Nicholas D.J.D., Nason A.J. (1954). Molybdenum and nitrate reductase. II. Molybdenum as a constituent of nitrate reductase. J. Biol. Chem..

[6] Cohen H.J., Drew R.T., Johnson J.L., Rajagopalan K.V. (1973). Molecular basis of the biological function of molybdenum: The relationship between sulfite oxidase and the acute toxicity of bisulfite and SO_2_. Proc. Natl. Acad. Sci. USA.

[7] Hille R., Hall J., Basu P. (2014). The mononuclear molybdenum enzymes. Chem. Rev..

[8] Magalon A., Fedor J.G., Walburger A., Weiner J.H. (2011). Molybdenum enzymes in bacteria and their maturation. Coord. Chem. Rev..

[9] Mendel R.R., Schwarz G. (2023). The History of Animal and Plant Sulfite Oxidase—A Personal View. Molecules.

[10] Hille R. (2023). Xanthine Oxidase—A Personal History. Molecules.

[11] Moura J.J.G. (2023). The History of *Desulfovibrio gigas* Aldehyde Oxidoreductase—A Personal View. Molecules.

[12] Seo M., Peeters A.J., Koiwai H., Oritani T., Marion-Poll A., Zeevaart J.A., Koornneef M., Kamiya Y., Koshiba T. (2000). The Arabidopsis aldehyde oxidase 3 (AAO3) gene product catalyzes the final step in abscisic acid biosynthesis in leaves. Proc. Natl. Acad. Sci. USA.

[13] Campbell W.H., Kinghorn K.R. (1990). Functional domains of assimilatory nitrate reductases and nitrite reductases. Trends Biochem. Sci..

[14] Clement B., Struwe M.A. (2023). The History of mARC. Molecules.

[15] Magalon A. (2023). History of Maturation of Prokaryotic Molybdoenzymes-A Personal View. Molecules.

[16] Bevers L.E., Hagedoorn P.-L., Hagen W.R. (2009). The bioinorganic chemistry of tungsten. Coord. Chem. Rev..

[17] Cove D.J., Pateman J.A. (1963). Independently segregating genetic loci concerned with nitrate reductase activity in *Aspergillus nidulans*. Nature.

[18] Pateman J.A., Cove D.J., Rever B.M., Roberts D.B. (1964). A common cofactor for nitrate reductase and xanthine dehydrogenase wich also regulates the synthesis of nitrate reductase. Nature.

[19] Mendel R.R., Müller A.J. (1976). A common genetic determinant of xanthine dehydrogenase and nitrate reductase in *Nicotiana tabacum*. Biochem. Physiol. Pflanz..

[20] Warner C.K., Finnerty V. (1981). Molybdenum hydroxylases in Drosophila. II. Molybdenum cofactor in xanthine dehydrogenase, aldehyde oxidase and pyridoxal oxidase. Mol. Gen. Genet..

[21] Smedley P.L., Kinniburgh D.G. (2017). Molybdenum in natural waters: A review of occurrence, distributions and controls. Appl. Geochem..

[22] Ketchum P.A., Cambier H.Y., Frazier W.A., Madansky C.H., Nason A. (1970). In vitro assembly of Neurospora assimilatory nitrate reductase from protein subunits of a Neurospora mutant and the xanthine oxidizing or aldehyde oxidase systems of higher animals. Proc. Natl. Acad. Sci. USA.

[23] Nason A., Antoine A.D., Ketchum P.A., Frazier W.A., Lee D.K. (1970). Formation of assimilatory nitrate reductase by in vitro inter-cistronic complementation in *Neurospora crassa*. Proc. Natl. Acad. Sci. USA.

[24] Nason A., Lee K.Y., Pan S.S., Ketchum P.A., Lamberti A., DeVries J. (1971). Invitro formation of assimilatory reduced nicotinamide adenine dinucleotide phosphate: Nitrate reductase from a Neurospora mutant and a component of molybdenum-enzymes. Proc. Natl. Acad. Sci. USA.

[25] Pienkos P.T., Shah V.K., Brill W.J. (1977). Molybdenum cofactors from molybdoenzymes and in vitro reconstitution of nitrogenase and nitrate reductase. Proc. Natl. Acad. Sci. USA.

[26] Johnson J.L., Hainline B.E., Rajagopalan K.V. (1980). Characterization of the molybdenum cofactor of sulfite oxidase, xanthine, oxidase, and nitrate reductase. Identification of a pteridine as a structural component. J. Biol. Chem..

[27] Johnson J.L., Rajagopalan K.V. (1982). Structural and metabolic relationship between the molybdenum cofactor and urothione. Proc. Natl. Acad. Sci. USA.

[28] Romao M.J., Archer M., Moura I., Moura J.J., LeGall J., Engh R., Schneider M., Hof P., Huber R. (1995). Crystal structure of the xanthine oxidase-related aldehyde oxido-reductase from *D. gigas*. Science.

[29] Fischer K., Barbier G.G., Hecht H.-J., Mendel R.R., Campbell W.H., Schwarz G. (2005). Crystal Structure of the Yeast Nitrate Reductase Molybdenum Domain Provides Insight into Eukaryotic Nitrate Assimilation. Plant Cell.

[30] Kisker C., Schindelin H., Pacheco A., Wehbi W.A., Garrett R.M., Rajagopalan K.V., Enemark J.H., Rees D.C. (1997). Molecular basis of sulfite oxidase deficiency from the structure of sulfite oxidase. Cell.

[31] Kisker C., Schindelin H., Rees D.C. (1997). Molybdenum-cofactor-containing enzymes: Structure and mechanism. Annu. Rev. Biochem..

[32] Cove D.J. (1979). Genetic studies of nitrate assimilation in *Aspergillus nidulans*. Biol. Rev. Camb. Philos. Soc..

[33] Mendel R.R., Alikulov Z.A., Lvov N.P., Müller A.J. (1981). Presence of the molybdenum-cofactor in nitrate reductase-deficient mutant cell lines of *Nicotiana Tabacum*. Mol. Gen. Genet..

[34] Tomsett A.B., Garrett R.H. (1980). The isolation and characterization of mutants defective in nitrate assimilation in *Neurospora Crassa*. Genetics.

[35] Rajagopalan K.V., Johnson J.L. (1992). The pterin molybdenum cofactors. J. Biol. Chem..

[36] Mendel R.R., Gresshoff P.M. (1992). The plant molybdenum cofactor (MoCo)—its biochemical and molecular genetics. Plant Biotechnology and Development—Current Topics in Plant Molecular Biology.

[37] Blattner F.R., Plunkett G., Bloch C.A., Perna N.T., Burland V., Riley M., Collado-Vides J., Glasner J.D., Rode C.K., Mayhew G.F. (1997). The complete genome sequence of *Escherichia coli* K-12. Science.

[38] Venter J.C. (2000). The sequence of the human genome. Science.

[39] Copenhaver G.P. (2000). Arabidopsis Genome Initiative. Analysis of the genome sequence of the flowering plant *Arabidopsis thaliana*. Nature.

[40] Mendel R.R., Müller A. (1979). Nitrate reductase-deficient mutant cell lines of *Nicotiana tabacum*. Further biochemical characterization. Mol. Gen. Genet. MGG.

[41] Stallmeyer B., Nerlich A., Schiemann J., Brinkmann H., Mendel R.R. (1995). Molybdenum co-factor biosynthesis: The *Arabidopsis thaliana* cDNA cnx1 encodes a multifunctional two-domain protein homologous to a mammalian neuroprotein, the insect protein Cinnamon and three *Escherichia coli* proteins. Plant J..

[42] Caboche M., Campbell W.H., Crawford N.M., Fernandez E., Kleinhofs A., Ida S., Mendel R.R., Amato T., Rothstein S., Wray J. (1994). Genes involved in nitrogen assimilation. Plant Mol. Biol. Rep..

[43] Mendel R.R., Kruse T. (2012). Cell biology of molybdenum in plants and humans. Biochim. Biophys. Acta.

[44] Schwarz G., Boxer D.H., Mendel R.R., Pfleiderer W., Rokos H. (1997). The plant protein Cnx1 binds molybdopterin and is involved in the last step of molybdenum cofactor biosynthesis. Chemistry and Biology of Pteridines and Folates.

[45] Schwarz G., Boxer D.H., Mendel R.R. (1997). Molybdenum cofactor biosynthesis. The plant protein Cnx1 binds molybdopterin with high affinity. J. Biol. Chem..

[46] Llamas A., Kalakoutskii K.L. (2008). Molybdenum cofactor amounts in *Chlamydomonas reinhardtii* depend on the Nit5 gene function related to molybdate transport. Plant Cell Environ..

[47] Fritschy J.M., Harvey R.J., Schwarz G. (2008). Gephyrin: Where do we stand, where do we go?. Trends Neurosci..

[48] Warnhoff K., Ruvkun G. (2019). Molybdenum cofactor transfer from bacteria to nematode mediates sulfite detoxification. Nat. Chem. Biol..

[49] Oliphant K.D., Rabenow M., Hohtanz L., Mendel R.R. (2022). The *Neurospora crassa* molybdate transporter: Characterizing a novel transporter homologous to the plant MOT1 family. Fungal Genet. Biol..

[50] Schwarz G., Schrader N., Mendel R.R., Hecht H.J., Schindelin H. (2001). Crystal structures of human gephyrin and plant Cnx1 G domains: Comparative analysis and functional implications. J. Mol. Biol..

[51] Liu M.T., Wuebbens M.M., Rajagopalan K.V., Schindelin H. (2000). Crystal structure of the gephyrin-related molybdenum cofactor biosynthesis protein MogA from Escherichia coli. J. Biol. Chem..

[52] Kuper J., Llamas A., Hecht H.J., Mendel R.R., Schwarz G. (2004). Structure of the molybdopterin-bound Cnx1G domain links molybdenum and copper metabolism. Nature.

[53] Llamas A., Otte T., Multhaup G., Mendel R.R., Schwarz G. (2006). The Mechanism of nucleotide-assisted molybdenum insertion into molybdopterin. A novel route toward metal cofactor assembly. J. Biol. Chem..

[54] Krausze J., Probst C., Curth U., Reichelt J., Saha S., Schafflick D., Heinz D.W., Mendel R.R., Kruse T. (2017). Dimerization of the plant molybdenum insertase Cnx1E is required for synthesis of the molybdenum cofactor. Biochem. J..

[55] Xiang S., Nichols J., Rajagopalan K.V., Schindelin H. (2001). The Crystal Structure of *Escherichia coli* MoeA and Its Relationship to the Multifunctional Protein Gephyrin. Structure.

[56] Krausze J., Hercher T.W., Zwerschke D., Kirk M.L., Blankenfeldt W., Mendel R.R., Kruse T. (2018). The functional principle of eukaryotic molybdenum insertases. Biochem. J..

[57] Probst C., Yang J., Krausze J., Hercher T.W., Richers C.P., Spatzal T., Kc K., Giles L.J., Rees D.C., Mendel R.R. (2021). Mechanism of molybdate insertion into pterin-based molybdenum cofactors. Nat. Chem..

[58] Kruse T. (2022). Function of Molybdenum Insertases. Molecules.

[59] Schrag J.D., Huang W., Sivaraman J., Smith C., Plamondon J., Larocque R., Matte A., Cygler M. (2001). The crystal structure of *Escherichia coli* MoeA, a protein from the molybdopterin synthesis pathway. J. Mol. Biol..

[60] Hercher T.W., Krausze J., Hoffmeister S., Zwerschke D., Lindel T., Blankenfeldt W., Mendel R.R., Kruse T. (2020). Insights into the Cnx1E catalyzed MPT-AMP hydrolysis. Biosci. Rep..

[61] Gabelli S.B., Bianchet M.A., Ohnishi Y., Ichikawa Y., Bessman M.J., Amzel L.M. (2002). Mechanism of the *Escherichia coli* ADP-ribose pyrophosphatase, a Nudix hydrolase. Biochemistry.

[62] Kirsch J., Wolters I., Triller A., Betz H. (1993). Gephyrin antisense oligonucleotides prevent glycine receptor clustering in spinal neurons [see comments]. Nature.

[63] Kirsch J., Betz H. (1995). The Postsynaptic Localization of the Glycine Receptor-Associated Protein Gephyrin Is Regulated by the Cytoskeleton. J. Neurosci..

[64] Stallmeyer B., Schwarz G., Schulze J., Nerlich A., Reiss J., Kirsch J., Mendel R.R. (1999). The neurotransmitter receptor-anchoring protein gephyrin reconstitutes molybdenum cofactor biosynthesis in bacteria, plants, and mammalian cells. Proc. Natl. Acad. Sci. USA.

[65] Feng G., Tintrup H., Kirsch J., Nichol M.C., Kuhse J., Betz H., Sanes J.R. (1998). Dual requirement for gephyrin in glycine receptor clustering and molybdoenzyme activity [see comments]. Science.

[66] Kamdar K.P., Primus J.P., Shelton M.E., Archangeli L.L., Wittle A.E., Finnerty V. (1997). Structure of the molybdenum cofactor genes in Drosophila. Biochem. Soc. Trans..

[67] Probst C., Ringel P., Boysen V., Wirsing L., Alexander M.M., Mendel R.R., Kruse T. (2014). Genetic characterization of the *Neurospora crassa* molybdenum cofactor biosynthesis. Fungal Genet. Biol..

[68] Dejanovic B., Semtner M., Ebert S., Lamkemeyer T., Neuser F., Luscher B., Meier J.C., Schwarz G. (2014). Palmitoylation of gephyrin controls receptor clustering and plasticity of GABAergic synapses. PLoS Biol..

[69] Schwarz G., Schulze J., Bittner F., Eilers T., Kuper J., Bollmann G., Nerlich A., Brinkmann H., Mendel R.R. (2000). The molybdenum cofactor biosynthetic protein Cnx1 complements molybdate- repairable mutants, transfers molybdenum to the metal binding pterin, and is associated with the cytoskeleton. Plant Cell.

[70] Kaufholdt D.B.C., Bikker R., Burkart V., Dudek C.A., von Pein L., Rothkegel M., Mendel R.R., Hänsch R. (2016). The molybdenum cofactor biosynthesis complex interacts with actin filaments via molybdenum insertase Cnx1 as anchor protein in *Arabidopsis thaliana*. Plant Sci..

[71] Kaufholdt D., Gehl C., Geisler M., Jeske O., Voedisch S., Ratke C., Bollhoner B., Mendel R.R., Hansch R. (2013). Visualization and quantification of protein interactions in the biosynthetic pathway of molybdenum cofactor in *Arabidopsis thaliana*. J. Exp. Bot..

[72] Minner-Meinen R., Weber J.N., Kistner S., Meyfarth P., Saudhof M., van den Hout L., Schulze J., Mendel R.R., Hansch R., Kaufholdt D. (2022). Physiological Importance of Molybdate Transporter Family 1 in Feeding the Molybdenum Cofactor Biosynthesis Pathway in *Arabidopsis thaliana*. Molecules.

[73] Gasber A., Klaumann S., Trentmann O., Trampczynska A., Clemens S., Schneider S., Sauer N., Feifer I., Bittner F., Mendel R.R. (2011). Identification of an *Arabidopsis* solute carrier critical for intracellular transport and inter-organ allocation of molybdate. Plant Biol..

[74] Tejada-Jimenez M., Galvan A., Fernandez E. (2011). Algae and humans share a molybdate transporter. Proc. Natl. Acad. Sci. USA.

[75] Weber J.N., Minner-Meinen R., Kaufholdt D. (2023). The Mechanisms of Molybdate Distribution and Homeostasis with Special Focus on the Model Plant *Arabidopsis thaliana*. Molecules.

[76] Oliphant K.D., Karger M., Nakanishi Y., Mendel R.R. (2022). Precise Quantification of Molybdate In Vitro by the FRET-Based Nanosensor ‘MolyProbe’. Molecules.

[77] Weiss M.C., Preiner M., Xavier J.C., Zimorski V., Martin W.F. (2018). The last universal common ancestor between ancient Earth chemistry and the onset of genetics. PLoS Genet..

[78] Zhang Y., Ying H., Xu Y. (2019). Comparative genomics and metagenomics of the metallomes. Metallomics.

[79] Zhang Y., Gladyshev V.N. (2008). Molybdoproteomes and evolution of molybdenum utilization. J. Mol. Biol..

[80] Lake M.W., Wuebbens M.M., Rajagopalan K.V., Schindelin H. (2001). Mechanism of ubiquitin activation revealed by the structure of a bacterial MoeB-MoaD complex. Nature.

[81] Mayr S.J., Mendel R.R., Schwarz G. (2021). Molybdenum cofactor biology, evolution and deficiency. Biochim. Biophys. Acta Mol. Cell Res..

[82] Oliphant K.D., Fettig R.R., Snoozy J., Mendel R.R., Warnhoff K. (2023). Obtaining the necessary molybdenum cofactor for sulfite oxidase activity in the nematode *Caenorhabditis elegans* surprisingly involves a dietary source. J. Biol. Chem..

